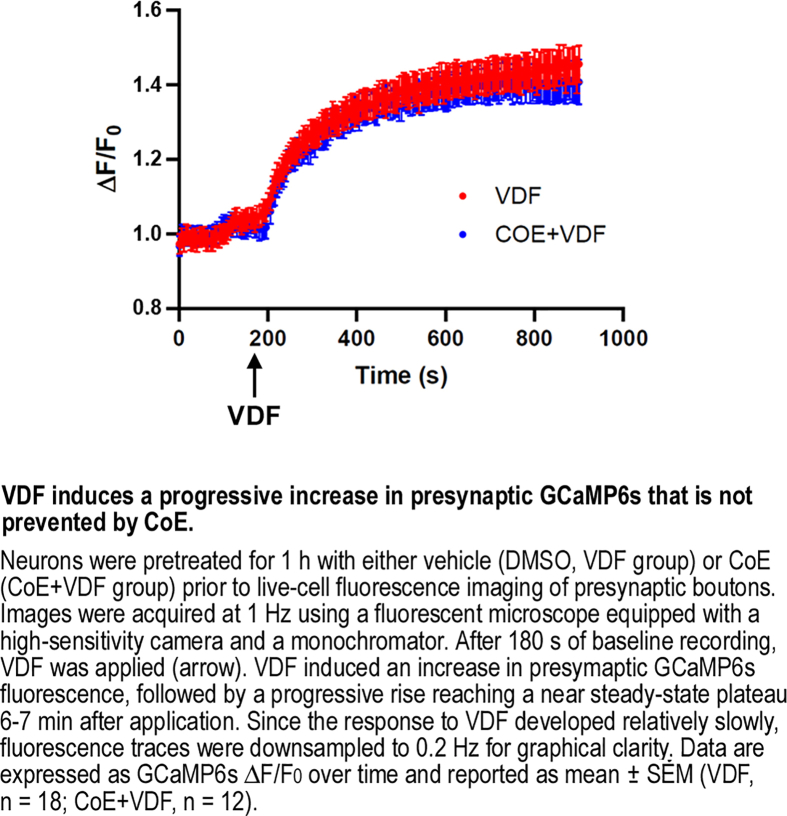# The PDE5 inhibitor vardenafil enhances glutamatergic transmission through amyloid-beta and cellular prion protein

**DOI:** 10.1016/j.neurot.2026.e00941

**Published:** 2026-06-07

**Authors:** Md Abdullah A. Kafi, Mario Passalacqua, Viviana Villa, Stefano Thellung, Alessandro Corsaro, Raffaella Grimaldi, Elena Spatola, Tullio Florio, Pietro Baldelli, Roberta Ricciarelli

**Affiliations:** a Department of Experimental Medicine, University of Genoa, Via L.B. Alberti 2, 16132 Genoa, Italy; b Center for the Promotion of the 3Rs Principles in Teaching & Research (Centro 3R), Università di Pisa, Largo Lucio Lazzarino 1, 56122 Pisa, Italy; c Department of Internal Medicine, University of Genoa, Viale Benedetto XV 6, 16132 Genoa, Italy; d IRCCS Azienda Ospedaliera Metropolitana, Largo Benzi 10, 16132 Genoa, Italy

**Keywords:** Alzheimer's disease, Erectile dysfunction drugs, Phosphodiesterase 5 inhibitor, Cyclic guanosine monophosphate (cGMP), Miniature excitatory postsynaptic current (mEPSC), Synaptic plasticity

## Abstract

Memory and synaptic plasticity are regulated by cyclic guanosine monophosphate (cGMP) signaling. Phosphodiesterase 5 (PDE5) inhibitors, such as vardenafil (VDF), elevate intracellular cGMP levels and represent potential therapeutics for cognitive disorders, including Alzheimer's disease (AD). However, the signaling pathways linking PDE5 inhibition to excitatory synaptic transmission remain incompletely defined. In particular, whether PDE5 inhibition engages amyloid-β (Aβ)-dependent mechanisms to regulate glutamatergic synapses remains unclear. Using biochemical, immunocytochemical, and electrophysiological approaches in neuronal systems, we show that VDF activates an Aβ-cellular prion protein (PrP^C^)-dependent pathway that enhances presynaptic glutamatergic function. Specifically, VDF increases Aβ levels, leading to a marked reduction in PrP^C^ surface exposure, an effect prevented by blocking Aβ production. We further demonstrate that PrP^C^ facilitates Aβ internalization, supporting a dynamic coupling between Aβ and PrP^C^ trafficking. Functionally, VDF selectively augments presynaptic excitatory transmission, as indicated by increased VGLUT1 puncta density and elevated miniature excitatory postsynaptic current (mEPSC) frequency, without changes in mEPSC amplitude. Disrupting Aβ-PrP^C^ interaction abolishes these presynaptic effects, establishing this axis as a required mediator of the synaptic response to VDF. Together, these findings identify a functional link between PDE5 inhibition and Aβ-PrP^C^-dependent modulation of presynaptic glutamatergic transmission, expanding the framework of Aβ-PrP^C^ signaling beyond neurotoxicity and highlighting its context-dependent role in synaptic regulation.

## Introduction

Cyclic guanosine monophosphate (cGMP) is a key second messenger that regulates multiple aspects of neuronal function [1]. Intracellular cGMP levels are tightly controlled by phosphodiesterases (PDEs), a large family of enzymes that includes PDE5, which selectively hydrolyzes cGMP [2]. Pharmacological inhibition of PDE5 is therefore widely used to modulate cGMP-dependent signaling pathways [3].

In the nervous system, PDE5 inhibitors have been reported to influence synaptic function [4,5]. However, the molecular mechanisms linking PDE5 inhibition to specific changes in excitatory synaptic transmission remain incompletely defined.

We previously showed that pharmacological inhibition of PDE5 with vardenafil (VDF) increases amyloid-β (Aβ) production in neuronal systems, identifying Aβ as a downstream effector of cGMP pathway activation [6,7]. Aβ peptides are generated by sequential cleavage of the amyloid precursor protein (APP) by β- and γ-secretases. While elevated levels of Aβ are implicated in Alzheimer's disease (AD) pathology, accumulating evidence indicates that, at physiological concentrations, Aβ contributes to the regulation of synaptic processes [8]. Despite these observations, the cellular targets and signaling pathways through which PDE5 inhibition-induced Aβ production modulates synaptic function remain unclear.

Soluble Aβ oligomers (Aβo) bind with high affinity to the cellular prion protein (PrP^C^) on the neuronal surface [9,10]. The Aβ-PrP^C^ interaction has been extensively studied in the context of neurotoxicity [[11], [12], [13]], yet its potential role in physiological synaptic modulation is less understood. Moreover, whether pharmacological manipulation of PDE5 engages this axis to regulate basal glutamatergic transmission has not been directly investigated.

Here, we examined whether VDF-induced Aβ production functionally couples to PrP^C^ signaling to modulate excitatory synaptic transmission. Our results indicate that PrP^C^ is involved in the cellular uptake of Aβ and that VDF reduces surface PrP^C^ expression in a γ-secretase-dependent manner. Notably, VDF enhances presynaptic glutamatergic function. Specifically, the increase in miniature excitatory postsynaptic current (mEPSC) frequency and VGLUT1-positive puncta density requires Aβ-PrP^C^ interaction, whereas postsynaptic AMPA receptor–mediated responses remain unchanged.

## Materials and Methods

### Cell lines and treatments

Mouse Neuro-2a (N2a) cells and N2a stably expressing wild-type human APP695 were obtained and grown as already reported [14]. Vardenafil (Merck, Darmstadt, Germany) and Compound E (Adipogen AG, Liestal, Switzerland) were dissolved in dimethyl sulfoxide (DMSO) and stored at −20 °C until use. FITC-labeled Aβ_42_ (GenicBio Limited, Shanghai, China) peptides were prepared essentially as previously described [15,16]. Briefly, lyophilized peptide was resuspended in 1,1,1,3,3,3-hexafluoro-2-propanol (HFIP) and aliquoted. The solvent was then allowed to evaporate, and the resulting clear peptide films were stored at −20 °C. Before use, HFIP-treated aliquots were resuspended in DMSO, sonicated, diluted in culture medium to 1 mM, and incubated at 4 °C for 24 h. Immunoblot analysis was performed on samples of this preparation, which revealed the presence of Aβ monomers and oligomers (Supplementary Fig. 1).

### Primary hippocampal neurons

Primary cultures of hippocampal neurons were prepared from wild type C57BL6/J mice of either sex at embryonic day 17 (E17), as previously described [17]. In brief, mice were sacrificed by CO2 inhalation, and embryos were removed immediately by caesarean section under a dissecting stereomicroscope. Hippocampi were dissociated by enzymatic digestion in 0.125% Trypsin for 20 min at 37 °C and then triturated with a fire-polished Pasteur pipette. Dissociated neurons were plated at low-density (100 cells/mm^2^) on Petri dishes previously pretreated with poly-l-lysine (0.1 mg/ml), and maintained in a culture medium consisting of Neurobasal, B-27 (1:50 v/v), glutamine (1% w/v), penicillin-streptomycin 1% (all from Invitrogen). All the experiments were performed in 13–15 days in vitro (DIV) neurons. All procedures were conducted in accordance with the guidelines of the National Council on Animal Care and were approved by the Animal Care Committee of the University of Genova.

### RNA interference

ON-TARGET plus Mouse Prnp siRNA SMART pool and Accell Non-targeting siRNA Pool were purchased from Dharmacon (Lafayette, CO, USA). Transfections were performed according to the manufacturer's instructions and silencing efficiency was verified by immunoblotting.

### Immunoblot analysis

Immunoblots were done according to standard methods, using the following mouse antibodies: anti-PrP^C^ (clone SAF32; Bertin Technologies SAS, Montigny-le-Bretonneux, FR), anti-β-actin and anti-Na^+^/K^+^ ATPase (Merck). Anti-mouse secondary antibody coupled to horseradish peroxidase was purchased from GE Healthcare (Milan, IT) Proteins were visualized with an enzyme-linked chemiluminescence detection kit according to the manufacturer's instructions (GE Healthcare). Chemiluminescence was monitored by exposure to films and signals were analyzed under non-saturating conditions with an image densitometer (Bio-Rad, Hercules, CA, USA).

### Enzyme-linked immunosorbent assay (ELISA)

At the end of cell treatments, conditioned media were collected, spun at 1000 *g* for 5 min to remove cell debris, and immediately subjected to ELISA specific for Aβ_42_ peptides (Human/Rat β Amyloid 42 ELISA kit, High Sensitive; Wako Chemicals, Neuss, DE) or PrP^C^ (Mouse Prion Protein ELISA kit; Reddot Biotech, Houston, TX, USA). Tests were carried out following the manufacturer's protocol, and Aβ and PrP^C^ concentrations were calculated according to the standard curves prepared on the respective ELISA plates.

### Surface PrP^C^ immunofluorescence

In N2a cell experiments, cells were grown overnight on culture slides, treated as indicated and then incubated for 1 h at 4 °C with the mouse anti-PrP^C^ SAF32 antibody (1:500). After fixation with ice-cold methanol, cells were labeled with a goat anti-mouse Alexa Fluor® 568 IgG (1:500; Thermo Fisher Scientific, Waltham, MA, USA) and analyzed using a Nikon AX-R confocal microscope equipped with a 60 × /1.42 N A. oil immersion objective.

In primary neurons experiments, cells were treated as indicated and incubated for 1 h at 4 °C with the mouse anti-PrP^C^ SAF32 antibody (1:500). After fixation with 2% paraformaldehyde and permeabilization with 0.1% Triton X-100, cells were incubated overnight at 4 °C with a rabbit anti-beta3tub antibody (1:800, Merck). Secondary antibodies used were Alexa Fluor® 488 chicken anti-rabbit IgG and Alexa Fluor® 568 goat anti-mouse IgG (1:500; Thermo Fisher Scientific). Images were acquired using a Nikon AX-R confocal microscope (20x/0.8 N A.). Analysis for both N2a cells and primary neurons was performed with FIJI ImageJ software.

### Cell surface biotinylation

At the end of treatments, N2a cells were surface biotinylated by incubation with Sulfo–NHS–SS–Biotin (Thermo Fisher Scientific) at 2 mg/ml in PBS for 30 min at 4 °C. Cells were then quenched with 100 mM glycine and lysed in 2 mM EGTA, 10 mM NaF, 1 mM Na3VO4, 5 mM EDTA, 150 mM NaCl, 50 mM Tris-HCl (pH 7.4), 1 mM phenylmethylsulphonyl fluoride, 1% protease inhibitor cocktail, and 1% sodium dodecyl sulfate. After centrifugation at 15,000 *g* for 10 min, supernatants were incubated for 2 h at 4 °C with NeutrAvidin™ Protein immobilized onto 6% crosslinked beaded agarose (Thermo Fisher Scientific), spun and subjected to SDS–PAGE and immunoblot analysis.

### Immunofluorescence of glutamatergic synapses

Primary hippocampal neurons were fixed with 4% paraformaldehyde/4% sucrose and permeabilized with 0.1% Triton X-100. Incubations with primary antibodies (guinea pig anti-VGLUT1, 1:500, Synaptic System; rabbit anti-beta3tub, 1:800, Merck) w ere performed overnight at 4 °C. Secondary antibodies were Alexa Fluor® 488 chicken anti-rabbit IgG and Alexa Fluor® 568 goat anti-guinea pig IgG (1:500; Thermo Fisher Scientific). Single optical sections were acquired using a Nikon AX-R confocal microscope and a 20x/0.8 N A. objective, with the confocal pinhole set at 1 AU. Sequential track for exiting green and red emitting dyes was performed. Image analysis was performed using FIJI ImageJ software. Labeling of VGLUT1 was measured using the freehand line tool with a width of 1 μm and a length of 60 μm. The fluorescence intensity profile was plotted as a function of the dendritic process length. Fluorescence background was measured by averaging the intensity of areas without cells and was subtracted from all fluorescence images. Only peaks exceeding an intensity threshold of three times the background level were considered.

### Electrophysiology

Whole-cell patch-clamp recordings were made at 20 kHz sampling rate and filtered at 1/5 of the acquisition rate with an 8-pole low-pass Bessel filter. Recordings with a leakage current > 100 pA or a series resistance >15 MΩ were discarded. Data acquisition was performed using the pClamp10 software (Molecular Device; LLC). Membrane potentials were not corrected for Donnan fluid junction potentials of 9 mV. All experiments were performed at room temperature (22–24 °C). Patch pipettes made of thin borosilicate glass were drawn and fire-polished to a final resistance of 3–4 MΩ when filled with internal standard solution. The internal solution was as follows (in mM): 126 K gluconate, 4 NaCl, 1 MgSO_4_, 0.02 CaCl_2_, 0.1 BAPTA, 15 glucose, 5 HEPES, 3 ATP, 0.1 GTP, pH 7.2 (with KOH). The external solution contained (in mM): 140 NaCl, 2 CaCl_2_, 1 MgCl_2_, 4 KCl, 10 glucose, 10 HEPES, pH 7.3 (with NaOH). Miniature Excitatory Postsynaptic Currents (mEPSCs) were recorded at a holding potential of −70 mV from low-density (160 cells/mm^2^) hippocampal neurons (13–15 DIV) in external solution supplemented with the following drugs: tetrodotoxin (TTX, 0.5 μM), bicuculline (30 μM) and D-AP5 (50 μM) to block spontaneous firing activity, GABAergic transmission and NMDA receptors, respectively. Amplitude, frequency, 10–90% rise and 50% decay time of mEPSCs were calculated using a peak detector function with a threshold amplitude set at 5 pA and a threshold area set at 50 ms∗pA. mEPSCs analysis was performed by using the Minianalysis (Synaptosoft, Leonia, NJ, USA) and the Prism (GraphPad Software Inc, San Diego, CA, USA) software.

### Presynaptic Ca^2+^ imaging with SyGCaMP6s

Live-cell imaging was performed as previously described [18]. Briefly, Mouse primary neuronal cultures were imaged at room temperature in standard external solution containing the following, in mM: 140 NaCl, 4 KCl, 2 CaCl_2_, 1 MgCl_2_, 10 d-glucose, and 10 HEPES, pH 7.3. VDF-dependent calcium changes were recorded in the presence of pharmacological blockers: tetrodotoxin (TTX; 1 μM), D-(−)-2-amino-5-phosphonopentanoic acid (D-AP5; 50 μM), and bicuculine (BIC; 30 μM). Cultures were transfected with SyGCaMP6s three days before the experiments and recorded at 14–16 DIV. Presynaptic boutons were imaged using a cooled charge-coupled device camera (CCD; ORCA-R2, Hamamatsu) mounted on an inverted microscope (Olympus IX71) equipped with a 40 ×, 1.35 NA oil-immersion objective. Illumination was provided by a TILL Photonics Polychrome IV monochromator. Images were acquired at 1 Hz with an integration time of 50 ms and an 8-bit acquisition depth. Image analysis was performed using ImageJ together with the Time Series Analyzer V3.0 plugin. Circular regions of interest (ROI) with a diameter of 3 μm were positioned on all boutons responding to VDF application. For local background subtraction, the fluorescence intensity of a paired ROI positioned within 10 μm of the corresponding bouton ROI was used. Calcium signals were quantified as ΔF/F_0_, where ΔF = F − F_0_, and F_0_ was calculated over a 180 s baseline period before VDF exposure.

### Statistical analysis

Results are expressed as mean ± standard error of the mean (SEM). Data were analyzed using 2-tailed Student *t*-test or one-way ANOVA followed by Dunnett post-hoc, as indicated. The level of significance was set at P < 0.05.

## Results

### Total expression of PrP^C^ and Aβ production are independent processes

Using N2a cells, we first investigated the potential correlation between PrP^C^ expression and Aβ peptide production. To this end, we induced transient knockdown of PrP^C^ with specific siRNAs and assessed Aβ release into the culture medium by ELISA. As shown in Fig. 1A, PrP^C^ knockdown had no impact on the amount of Aβ peptides released by the cells. To explore whether, conversely, variations in Aβ levels could influence the expression of PrP^C^, we treated the cells with the PDE5 inhibitor VDF, to induce Aβ production [6,7], and measured the total protein expression of PrP^C^ by immunoblotting. The VDF concentration and exposure times were selected based on our previous studies [6,7]. As expected, 5 h of VDF treatment significantly increased the amount of Aβ peptides released into the culture medium, however, this effect did not alter the total expression of PrP^C^ (Fig. 1B).

**Fig. 1:**
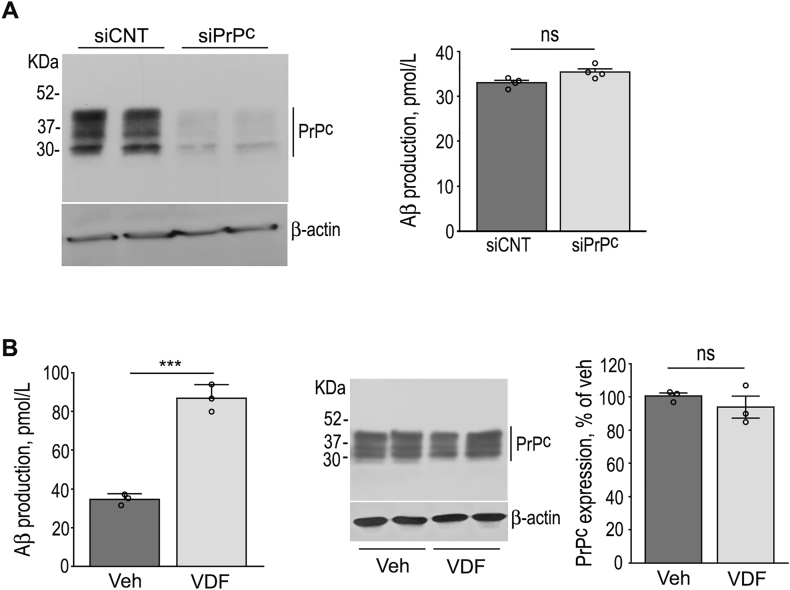
**Lack of correlation between PrP^C^ expression and Aβ_42_ production. (A)** PrP^C^ knockdown does not affect Aβ_42_ production. N2a cells were treated with PrP^C^ siRNA or control siRNA for 48 h and then washed and incubated in standard medium for 5 h. At the end of the incubation period, conditioned media were subjected to specific Aβ_42_ ELISA, and the cells were processed for total protein extraction followed by immunoblot analysis performed with the anti-PrP^C^ antibody SAF-32. **(B)** Induction of Aβ_42_ does not affect PrP^C^ expression. Cells were stimulated with 100 μM VDF or an equal volume of vehicle (Veh, DMSO) for 5 h and then processed for immunoblot analysis. Quantification of Aβ_42_ was performed on conditioned media by specific ELISA. Graphed data show mean ± SEM for at least 3 independent experiments. β-actin signal represents internal loading control. ns = not statistically different; ∗∗∗P < 0.001; Student *t*-test.

### VDF reduces PrP^C^ surface exposure by stimulating Aβ production

Given the established role of PrP^C^ as a receptor for Aβo [[9], [10], [11], [12]], we aimed to visualize their interaction at the cell surface using confocal microscopy. Following VDF treatment to increase the production and release of Aβ, N2a cells were processed for immunofluorescence labeling of surface PrP^C^ and Aβ. However, despite repeated attempts, we failed to detect the Aβ signal, regardless of the treatment conditions (Supplementary Fig. 2). Nonetheless, confocal analysis revealed a significant decrease in PrP^C^ immunofluorescence at the cell surface of VDF-treated samples compared with controls (−63.7 ± 1.17%) (Fig. 2A). To assess whether this effect was mediated by Aβ, cells were pretreated with Compound E (CoE), a selective γ-secretase inhibitor that blocks Aβ generation [19], prior to VDF stimulation. As shown in Fig. 2A, CoE abolished the VDF-induced reduction in PrP^C^ surface expression, indicating that Aβ is required for this effect.

**Fig. 2:**
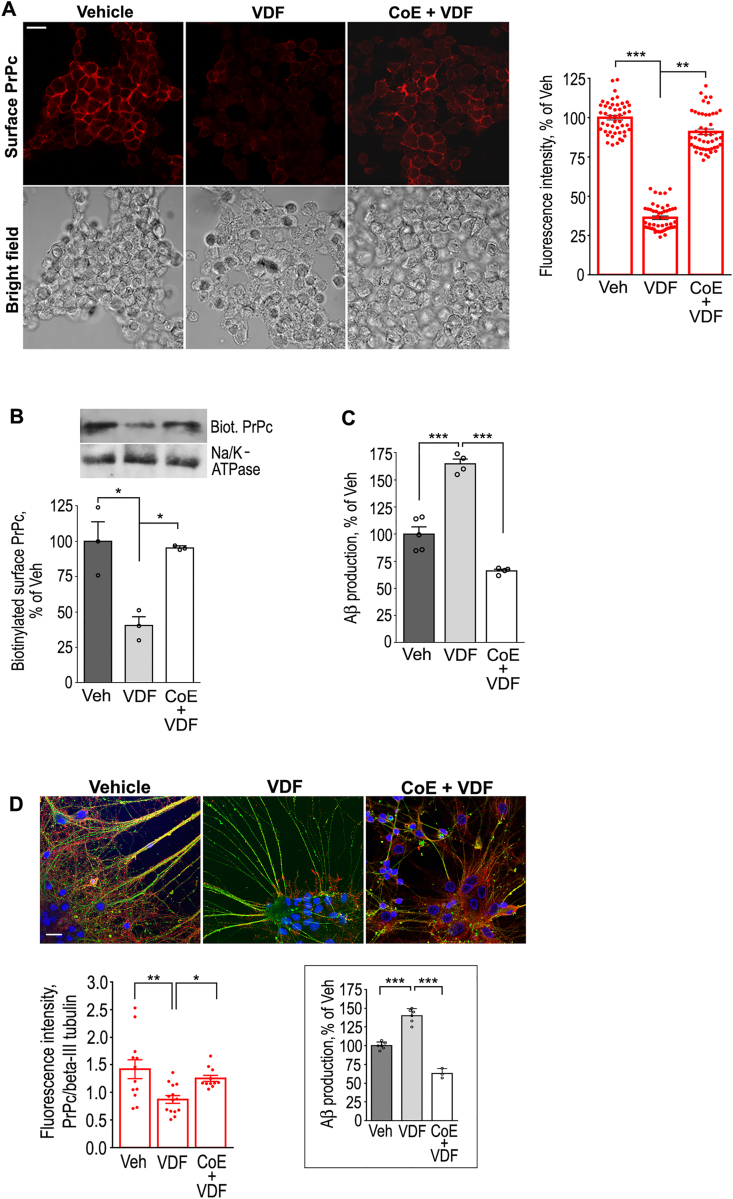
**VDF-induced reduction of PrP^C^ on the cell surface is reversed by inhibition of γ-secretase. (A)** Immunofluorescence analysis of PrP^C^ surface expression in N2a cells. Where indicated, cells were pretreated with the γ-secretase inhibitor CoE (1 μM) for 1 h and then stimulated with 100 μM VDF or an equal volume of vehicle (DMSO) for an additional 1 h. At the end of treatments, cells were incubated at 4 °C with the anti-PrP^C^ antibody SAF-32, fixed and labeled with the red-fluorescent secondary antibody Alexa Fluor® 568. Confocal microscopy images are representative of 3 independent experiments, the results of which were quantified using FIJI ImageJ software. Scale bar represents 20 μm. **(B)** Cell surface biotinylation. Cells were treated with CoE and/or VDF as in (A) and then processed for surface biotinylation followed by cell lysis and precipitation with NeutrAvidin® beads (see Material and Methods). Levels of PrP^C^ at the plasma membrane were determined by immunoblot (SAF-32 antibody). Na/K ATPase signal was used as a loading control. **(C)** Validation of the inhibitory effect of CoE on Aβ production. At the end of the cell treatments with VDF and/or CoE, carried out as in (A) and (B), the conditioned media were subjected to specific Aβ_42_ ELISA. **(D)** Immunofluorescence analysis of PrP^C^ surface expression in hippocampal primary neurons. Treatments were performed as described in (A). After fixation and permeabilization, neurons were immunostained for PrP^C^ (red fluorescence) and the neuron-specific marker beta-III tubulin (green fluorescence). Confocal microscopy images are representative of experiments performed on three independent primary culture preparations. Scale bar represents 20 μm. Fluorescence analysis for both N2a cells and primary neurons was performed with FIJI ImageJ software. *Inset:* validation of the inhibitory effect of CoE on Aβ production, performed as in (C). Bar graphs show mean ± SEM from at least three independent experiments (∗P < 0.05; ∗∗P < 0.01; ∗∗∗P < 0.001; one-way ANOVA, Dunnett post-test).

To substantiate these findings and exclude potential methodological artifacts, cells were treated with CoE and/or VDF and then processed for cell surface protein biotinylation. Immunoblot analysis of the biotin-labeled fractions confirmed that the amount of PrP^C^ expressed on the plasma membrane decreases after VDF treatment (−59.5 ± 6.15% vs control) and that the inhibition of Aβ production by CoE abrogates this effect (Fig. 2B). In parallel experiments, the efficacy of CoE in suppressing the production of Aβ was verified by specific Aβ-ELISA performed on cell-conditioned media (Fig. 2C).

To evaluate the physiological relevance of these observations, we next examined the effects of VDF in mature primary hippocampal neurons. Consistent with the findings in N2a cells, VDF treatment significantly decreased PrP^C^ surface expression, whereas CoE abrogated this effect (Fig. 2D). In the same experimental set, Aβ peptides levels in the conditioned media were quantified by specific Aβ-ELISA (Fig. 2D, *inset*).

**Fig. 3:**
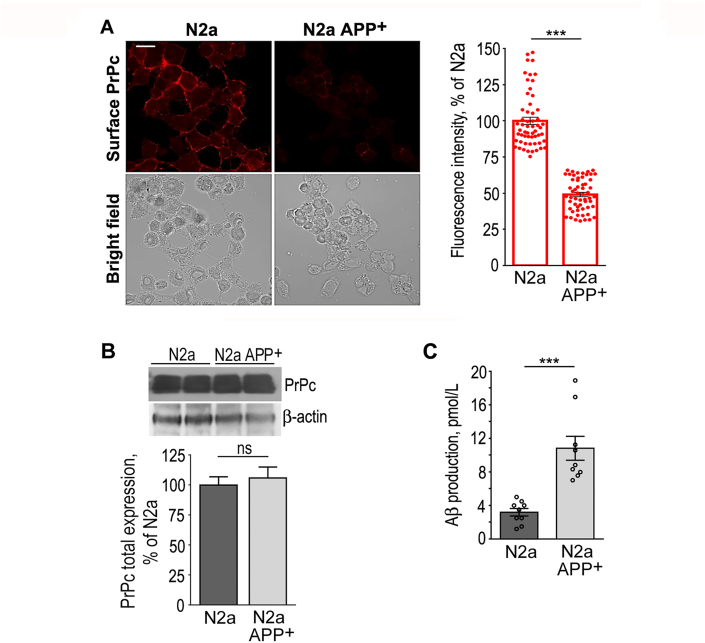
**PrP^C^ expression on the cells surface is affected by the overexpression of APP. (A)** Immunofluorescence of PrP^C^ on the cell surface of N2a wild type (N2a) and N2a overexpressing human APP (N2a APP^+^). Cells were incubated at 4 °C with the anti-PrP^C^ antibody SAF-32, as described in Materials and Methods, and then fixed and labeled with the red-fluorescent secondary antibody Alexa Fluor® 568. Fluorescence intensity of the images taken by confocal microscopy was quantified with FIJI ImageJ software. Scale bar represents 20 μM. **(B)** Total PrP^C^ expression in N2a and N2a APP^+^. Immunoblots of cell extracts were performed with SAF-32 anti-PrPc antibody. β-actin signal represents internal loading control. **(C)** Aβ production in N2a and N2a APP^+^ cells. An equal number of cells from both cell lines were plated. Twenty-four hour later, the culture medium was replaced with fresh medium, and after 1 h, Aβ_42_ levels in the conditioned medium were measured using a specific ELISA assay. Graphed data show mean ± SEM for at least 3 independent experiments. ∗∗∗P < 0.001; ns = not statistically different; Student *t*-test.

Collectively, these results indicate an inverse correlation between Aβ production and PrP^C^ expression at the neuronal plasma membrane. To further corroborate this correlation, we analyzed the surface expression of PrP^C^ in N2a cells overexpressing APP, which consequently produce higher levels of Aβ peptides (Fig. 3C). In agreement with the results obtained so far, APP-overexpressing cells displayed significantly reduced basal surface immunofluorescence of PrP^C^ compared with wild-type N2a (−51 ± 1.47%) (Fig. 3A), while total PrP^C^ protein levels remained unchanged (Fig. 3B).

### Role of PrP^C^ in Aβ internalization

**Fig. 4:**
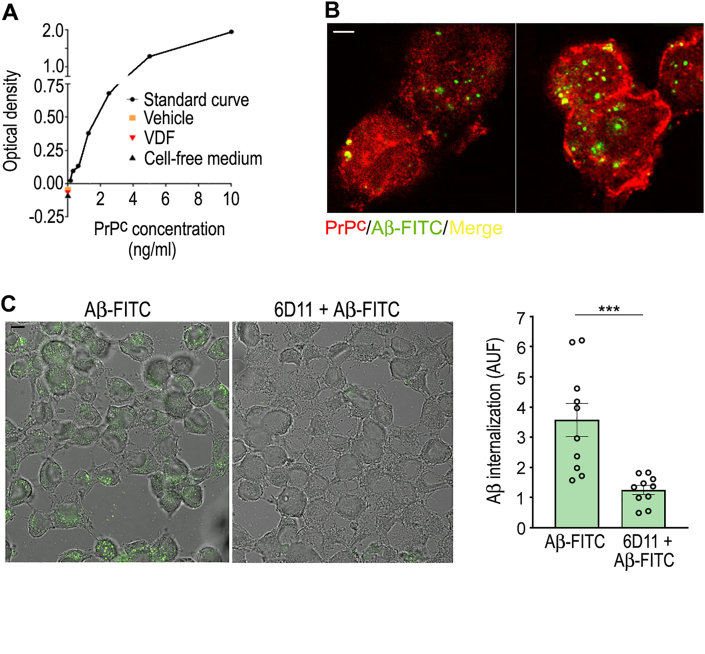
**Analysis of PrP^C^ localization after interaction with Aβ. (A)** Detection of PrP^C^ in conditioned media. N2a cells were treated for 16 h with 100 μM VDF or an equal volume of vehicle (DMSO). At the end of treatments, conditioned media were subjected to PrP^C^ ELISA. The graph shows the results of experiments repeated 3 times with substantially similar results. **(B)** Subcellular localization of PrP^C^ after exposure of cells to Aβ_42_ peptides labeled with FITC (Aβ-FITC). Cells were incubated for 1 h with 100 nM Aβ-FITC. At the end of the treatment, cells were fixed, permeabilized, incubated with the anti-PrP^C^ antibody SAF-32, and finally labeled with the red-fluorescent secondary antibody Alexa Fluor® 568. White scale bar = 10 μm. **(C)** The anti-PrP^C^ antibody 6D11 reduces the uptake of Aβ peptides. Where indicated, cells were pretreated with 6D11 (4 μg/ml) for 1 h and then incubated with 100 nM Aβ-FITC for an additional 1 h. At the end of treatments, cells were fixed and processed for confocal microscopy; black scale bar = 20 μm. Data are presented as mean ± SEM from three independent experiments. Each open circle represents the AUF of a randomly chosen field, normalized to cell number (∗∗∗P < 0.001; Student *t*-test).

To rule out the possibility that Aβ induces the shedding of PrP^C^ into the extracellular compartment, we performed specific PrP^C^-ELISA on the conditioned media of N2a cells treated with VDF. However, we did not detect measurable amounts of PrP^C^, even after 16 h of VDF exposure (Fig. 4A).

Since the endogenously produced Aβ was undetectable by confocal microscopy (Supplementary Fig. 2), likely due to very low concentration of Aβ released under physiological conditions, we used synthetic Aβ_42_ labeled with FITC (Aβ-FITC). We applied a standard procedure to generate an Aβ-FITC preparation that included Aβ monomers and oligomers (see Materials and Methods, and Supplementary Fig. 1). After 1 h of exposure to the Aβ-FITC preparation (100 nM, based on the molecular weight of the monomeric peptide), cells were washed extensively, fixed, permeabilized, and then processed for PrP^C^ immunofluorescence. Confocal microscopy analysis revealed that, under our experimental conditions, PrP^C^ and Aβ colocalize in close proximity to the plasma membrane but not within deeper intracellular compartments, which were, however, reached by Aβ-FITC (Fig. 4B). To assess the requirement of PrP^C^ in Aβ uptake, N2a cells were pretreated with the 6D11 anti-PrP^C^ monoclonal antibody, which acutely blocks Aβ binding [9], prior to exposure to the Aβ-FITC preparation. Under these conditions, 6D11 treatment significantly decreased Aβ-FITC uptake by 65% (Fig. 4C), supporting a role of PrP^C^ in mediating Aβ internalization.

### VDF increases glutamatergic synaptic density via Aβ peptides

These data are consistent with Aβ uptake via interaction with PrP^C^, although the functional relevance of this process for synaptic transmission remains unclear. Given that glutamatergic signaling is a major determinant of excitatory synaptic function, and that Aβ-PrP^C^ complexes have been reported to affect glutamatergic pathways [[11], [12], [13]], we investigated whether VDF-induced Aβ production modulates basal glutamatergic transmission in primary hippocampal neurons.

**Fig. 5:**
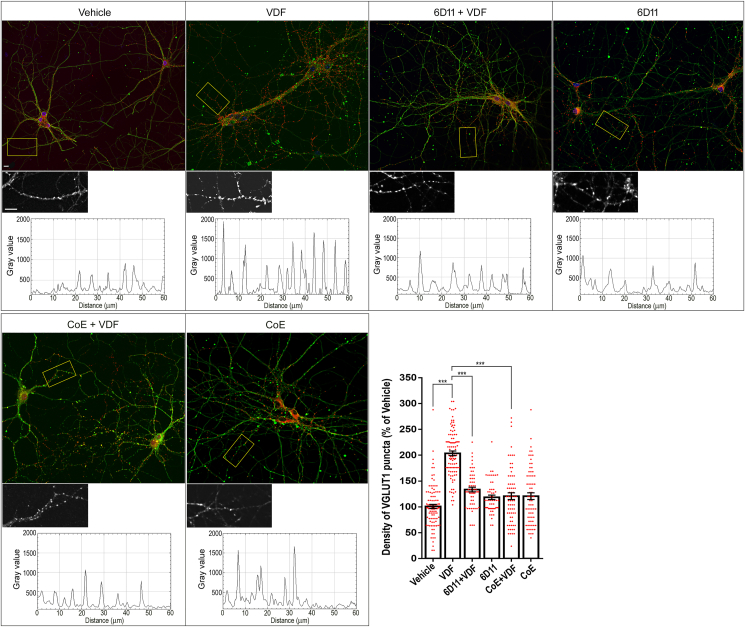
**VDF-induced increase in VGLUT1 puncta density requires Aβ-PrP^C^ interaction.** Where indicated, primary hippocampal neurons were pretreated with the anti-PrP^C^ antibody 6D11 or with the γ-secretase inhibitor CoE for 1 h and then incubated with 100 μM VDF or an equal volume of vehicle (DMSO) for an additional hour. At the end of treatments, neurons were fixed, permeabilized and immunostained for of VGLUT1 (red fluorescence) and the neuron-specific marker beta-III tubulin (green fluorescence). Scale bar: 10 μm. For each condition tested, a representative dendritic segment is outlined by a yellow rectangle and magnified in the grayscale inset (showing VGLUT1 fluorescence). The corresponding fluorescence intensity profile was obtained with FIJI ImageJ software, as described in the Materials and Methods section. Bar graph shows mean ± SEM of experiments performed three times on independent culture preparations, each performed in duplicate. ∗∗∗P < 0.001; one-way ANOVA, Dunnett post-test).

To evaluate the structural organization of excitatory presynaptic terminals under VDF stimulation, we quantified VGLUT1-immunoreactive puncta density along defined dendritic segments. VGLUT1 is a selective marker of glutamatergic presynaptic boutons. VDF treatment produced a significant increase in VGLUT1-positive puncta density (+103.6 ± 4.54% relative to control). This effect was markedly attenuated by pretreatment with either 6D11 or the γ-secretase inhibitor CoE, indicating that both Aβ production and Aβ–PrP^C^ interaction are required for the observed presynaptic changes (Fig. 5).

### PDE5 inhibition increases spontaneous glutamate release from presynaptic terminals

To determine whether the increase in VGLUT1-positive puncta was associated with functional changes in excitatory synaptic transmission, we performed whole-cell voltage-clamp recordings of miniature excitatory postsynaptic currents (mEPSCs) in primary hippocampal neurons.

**Fig. 6:**
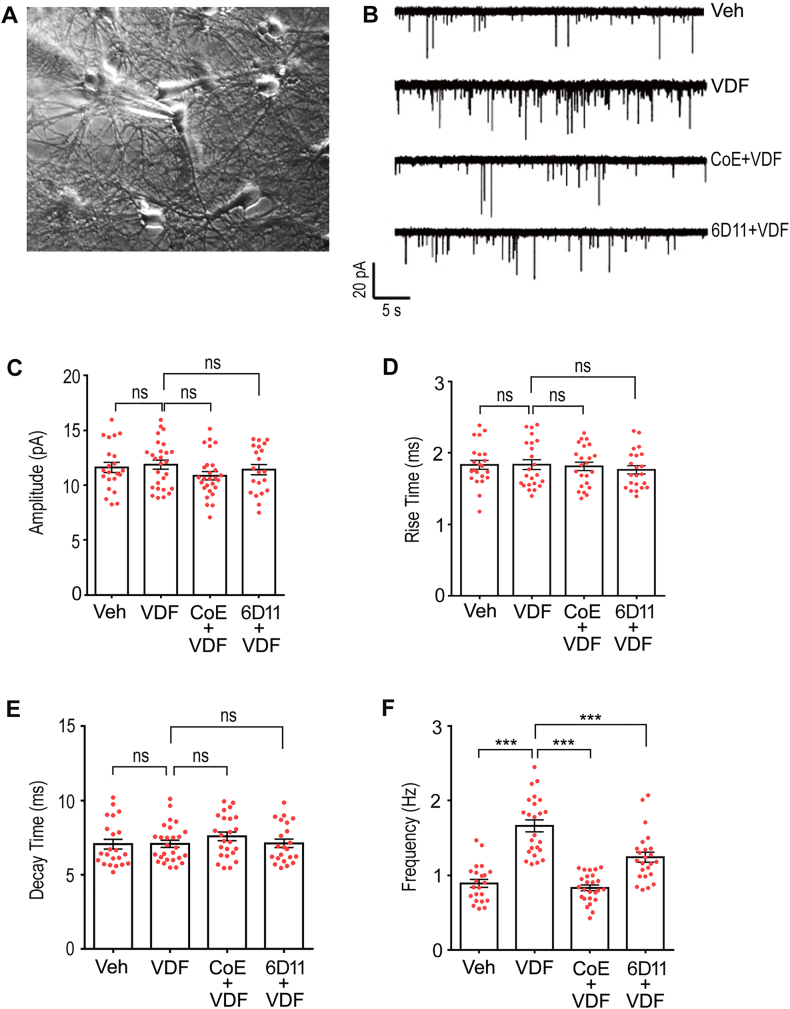
**VDF treatment increases the frequency of miniature EPSCs. (A)** Phase-contrast micrograph of a recording electrode positioned on a cultured hippocampal neuron. **(B)** Representative traces of mEPSCs recorded in low-density hippocampal neurons (13–15 DIV) treated with 100 μM VDF or an equal volume of vehicle (DMSO) for 1 h. Where indicated, neurons were pretreated with the γ-secretase inhibitor CoE or with the anti-PrP^C^ antibody 6D11. **(C)** Amplitude, (**D**) 10%–90% rise-time, (**E**) 50% decay-time and (**F**) frequency. Bar graphs show mean ± SEM from three independent neuronal preparations (n = 20–25). ∗∗∗P < 0.001; ns = not statistically different (one-way ANOVA, Dunnett post-test).

Recordings were obtained at −70 mV in the presence of tetrodotoxin (TTX) to block action potentials, bicuculline to inhibit GABAergic transmission, and AP-5 to isolate AMPA receptor–mediated events (Fig. 6). Under these conditions, mEPSCs reflect action potential–independent quantal glutamate release from presynaptic terminals.

VDF treatment did not significantly alter mEPSC amplitude or kinetics (Fig. 6C–E), indicating that postsynaptic AMPA receptor–mediated responses were not detectably affected. In contrast, VDF significantly increased mEPSC frequency (+86.32 ± 4.79% relative to control; Fig. 6F), consistent with an increase in the number and/or activity of functional glutamate release sites. Notably, pretreatment with the γ-secretase inhibitor CoE abolished the VDF-induced increase in mEPSC frequency, whereas 6D11 pretreatment reduced it by 54.47 ± 5.39% (Fig. 6F). These findings demonstrate that VDF-induced presynaptic enhancement requires Aβ production and is largely mediated by Aβ–PrP^C^ interaction.

Because the increased mEPSC frequency could reflect changes in presynaptic Ca2^+^ signaling, we monitored presynaptic Ca^2+^ dynamics using Synaptophysin-GCaMP6s, a genetically encoded Ca^2+^ indicator targeted to synaptic vesicles. VDF increased Synaptophysin-GCaMP6s fluorescence responses, indicating enhanced presynaptic Ca^2+^ dynamics (Supplementary Fig. 3). This effect was not prevented by CoE, suggesting that the VDF-induced increase in presynaptic Ca^2+^ responses does not require Aβ production.

## Discussion

In the present study, we identify a functional link between pharmacological PDE5 inhibition and presynaptic glutamatergic transmission mediated by Aβ-PrP^C^ interaction. Specifically, we show that VDF reduces surface PrP^C^ expression in an Aβ-dependent manner and enhances spontaneous excitatory transmission, as indicated by increased VGLUT1-positive puncta density and mEPSC frequency. Importantly, postsynaptic AMPA receptor-mediated parameters remain unchanged, indicating a selective presynaptic effect.

Previous studies have described complex and sometimes contradictory roles of PrP^C^ in Aβ biology. PrP^C^ has been suggested to influence APP processing, reducing the production of Aβ peptides [20]. However, our data do not support a repressive effect on Aβ production, consistent with more recent reports that also failed to detect an inhibitory role in amyloidogenic APP cleavage [21]. Instead, our findings indicate that PrP^C^ contributes to Aβ internalization, which may help explain earlier observations that PrP^C^-Aβ interactions elevate intracellular Aβ levels [22]. Notably, the reduction of surface PrP^C^ observed upon VDF treatment is most plausibly explained by receptor internalization, as total PrP^C^ levels remained unchanged and no increase in extracellular release was detected. Consistent with this interpretation, recent evidence has shown that PrP^C^-Aβ complexes can be internalized via caveolin-1-dependent pathways [23]. However, internalization of PrP^C^-Aβ complexes was not directly assessed under the experimental conditions used in the present study. Therefore, while our findings are consistent with a model in which Aβ binding promotes PrP^C^-Aβ complex internalization, additional studies will be required to define the trafficking pathways involved.

A key observation of this work is that VDF-induced enhancement of presynaptic glutamatergic transmission requires both Aβ production and Aβ-PrP^C^ interaction. The increase in mEPSC frequency, together with unchanged amplitude and kinetics, indicates that PDE5 inhibition selectively augments spontaneous presynaptic release without altering postsynaptic AMPA receptor responsiveness. This functional profile is consistent with an increase in the number and/or activity of glutamate release sites, in agreement with the observed increase in VGLUT1-positive puncta. Additional Synaptophysin-GCaMP6s imaging experiments further refine this interpretation. VDF increased presynaptic Ca^2+^ responses, but this effect was not prevented by inhibition of Aβ production (Supplementary Fig. 3). Thus, PDE5 inhibition appears to engage at least two partially dissociable presynaptic mechanisms: an Aβ–PrP^C^-independent component that enhances presynaptic Ca^2+^ dynamics, and an Aβ–PrP^C^-dependent component required for the increase in VGLUT1-positive puncta and mEPSC frequency. Because the VDF-induced increase in mEPSC frequency was abolished by CoE and markedly reduced by 6D11 despite preserved VDF-induced Ca2^+^ responses, the present data are consistent with the possibility that Aβ–PrP^C^ signaling acts downstream of, or in parallel with, Ca^2+^ entry to regulate functional release-site availability or synaptic vesicle release competence. However, this remains indirect, since vesicle fusion, readily releasable pool size, and the molecular components of the release machinery were not directly assesed in the present study.

Although Aβ-PrP^C^ complexes have been extensively studied in the context of neurotoxicity [9,24,25], particularly in association with glutamatergic dysfunction [[11], [12], [13],^26^], their role in regulating basal synaptic transmission has remained less defined. Our results indicate that this molecular interaction can contribute to physiological modulation of presynaptic function under conditions of pharmacological PDE5 inhibition. It should be noted that, although previous studies have implicated mGluR5 in Aβ-PrP^C^-dependent effects [26,27], our experimental design did not directly assess the involvement of mGluR5 or other metabotropic receptors. Consequently, any contribution of these pathways remains speculative and warrants further investigation.

Nevertheless, the observation that PDE5 inhibition, and the consequent increase in cGMP, enhances glutamatergic synapses presents an apparent paradox. While glutamate is essential for learning and memory, and VDF's ability to increase glutamatergic synaptic density likely underpins its cognitive-enhancing effects, excessive glutamate activity is associated with excitotoxicity, a key driver of neurodegeneration. A second, related paradox arises from the Aβ dependence of VDF's effects, raising the question of whether PDE5 inhibitors may alter Aβ homeostasis, another crucial factor in AD pathogenesis. These apparent contradictions may be reconciled by considering that Aβ, like glutamate, exhibits hormetic properties, exerting either neurotoxic or neurotrophic effects depending on its concentration [28]. Indeed, while elevated levels of Aβ are associated with well-documented detrimental effects, low concentrations have been shown to enhance synaptic plasticity and memory [15] and are essential for the viability of central neurons [29]. In this context, the increase in Aβ that we detected in our studies falls within the picomolar range, which has frequently been associated with physiological neuromodulatory actions [15,28,29].

At the same time, caution is warranted when interpreting the long-term implications of these observations. Our measurements were performed under acute experimental conditions and quantified soluble extracellular Aβ species, without directly assessing their aggregation state or long-term aggregation fate. Importantly, several studies have demonstrated that Aβ_42_ can undergo oligomerization and nucleation even at physiologically relevant concentrations, particularly in the presence of membranes, surfaces, or other aggregation-promoting microenvironments [[30], [31], [32]]. Consequently, although the picomolar Aβ levels observed in our experiments are compatible with a physiological role of soluble Aβ, the present data do not allow conclusions regarding whether sustained PDE5 inhibition could ultimately influence amyloid aggregation or plaque formation over longer time scales. It should also be noted that the concentration of VDF used in the present study (100 μM) was selected for mechanistic investigation and is substantially higher than clinically achievable plasma concentrations of PDE5 inhibitors. Accordingly, the translational implications of these findings should be interpreted with caution.

Within these limitations, our findings support the idea that VDF increases the availability of soluble Aβ species involved in physiological synaptic function. This effect may be particularly relevant under pathological conditions, where Aβ is sequestered into aggregated plaques, potentially disrupting its physiological role. In support of this view, PDE5 inhibitors have been shown to improve cognitive performance in AD animal models [[33], [34], [35]]. Moreover, insoluble amyloid plaques can be present in cognitively normal individuals, whereas reduced levels of soluble Aβ represent a consistent feature of AD [[36], [37], [38], [39]]. However, these findings should not be interpreted as evidence that chronic PDE5 inhibition is necessarily devoid of amyloidogenic consequences. Definitive assessment of the translational implications of PDE5 inhibition will require long-term in vivo studies directly examining amyloid plaque burden, soluble and insoluble Aβ species, and disease progression following sustained treatment.

Additional considerations should also be noted. First, we did not perform sex-stratified analyses in neuronal cultures, which represents a limitation of the present study. This is particularly relevant given emerging evidence for sex-dependent differences in Aβ-PrP^C^ signaling. Notably, Aβ-PrP^C^ complexes have been reported to be detectable in male but not female donors [40], suggesting differential regulation of this pathway across sexes. Moreover, the higher incidence of AD in women further highlights the importance of considering sex as a biological variable. While our findings provide insight into Aβ-PrP^C^-dependent modulation of synaptic function, future studies incorporating sex-stratified designs will be important to define potential sex-specific differences.

Second, while inhibition of γ-secretase effectively prevents Aβ production, off-target effects on additional substrates cannot be excluded. However, our experimental approaches support a role for Aβ beyond the contribution of other γ-secretase products. In particular, the results obtained using APP-overexpressing cells (Fig. 3) and the 6D11 antibody, which specifically disrupts the interaction between Aβ and PrP^C^ (Fig. 4, Fig. 5, Fig. 6), provide independent evidence reinforcing the involvement of Aβ, despite the known pleiotropic activity of γ-secretase.

Finally, the precise mechanism by which the Aβ-PrP^C^ complex enhances glutamatergic transmission remains to be fully characterized, including whether this effect requires Aβ internalization or is mediated by intracellular signaling triggered at the neuronal surface.

In summary, our findings demonstrate that PDE5 inhibition engages an Aβ-PrP^C^-dependent mechanism that selectively enhances spontaneous presynaptic glutamatergic transmission. These results extend current understanding of how pharmacological modulation of PDE5 intersects with amyloid biology and identify a previously underappreciated, context-dependent role for Aβ-PrP^C^ signaling in the regulation of basal excitatory synaptic function.

## Author contributions

RR: Conceptualization, Supervision, Data curation, Writing – original draft. TF: Resources, Writing – review & editing. PB: Supervision, Data curation, Resources, Writing – review & editing, Funding acquisition. MAK, MP, VV, AC, ST, RG, and ES: Methodology, Investigation, Formal analysis.

## Declaration of competing interest

The authors declare that they have no known competing financial interests or personal relationships that could have appeared to influence the work reported in this paper.

## Figures and Tables

**figs1:**
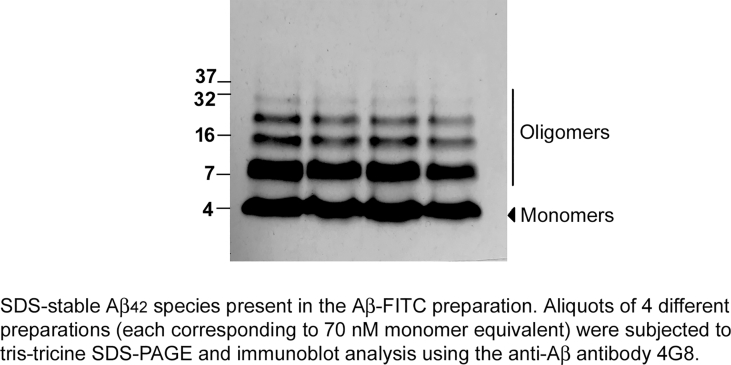


**figs2:**
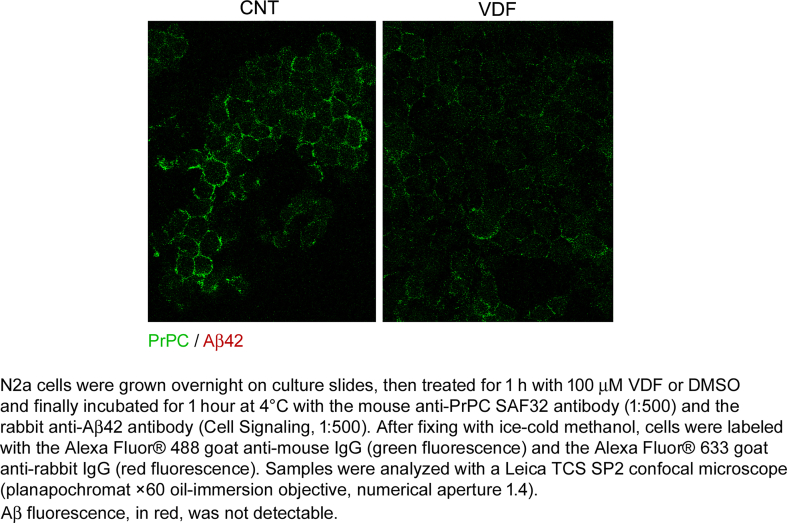


**figs3:**